# Moral Distress in the Everyday Life of an Intensivist

**DOI:** 10.3389/fped.2016.00091

**Published:** 2016-08-29

**Authors:** Daniel Garros

**Affiliations:** ^1^Pediatric Intensive Care Unit, Department of Pediatrics, 3A3 WC Mackenzie Health Sciences Centre, Stollery Children’s Hospital, University of Alberta, Edmonton, AB, Canada

**Keywords:** moral distress, ethics, medical, end-of-life care, decision-making, pediatric critical care medicine

A regular work day for intensivists can be emotionally draining, as we witness suffering, fear, pain, tragedies, unfair treatment of children, death…. We may experience the mental stress of dealing with nursing shortages, increasing family demands, and frustration related to interpersonal conflicts (e.g., between parents and specialists) among other issues (1). For the most part, we learn to manage this type of stress.

Several studies involving nearly every medical and surgical specialty indicate, however, that approximately one of every three physicians experiences burnout at any given time. Burnout is characterized by behaviors such as losing enthusiasm for work (emotional exhaustion), treating people as if they were objects (depersonalization), and having a sense that work is no longer meaningful (low personal accomplishment) (2).

Physicians, like other health-care professionals, can be at risk for another phenomenon, that of moral distress (MoD). This concept emerged in nursing ethics: “a challenge that arises when one has an ethical or moral judgment about care that differs from those who are in charge” (3). Thus, institutional constraints were seen as its key source (inadequate staffing, other professionals’ influence, family or patient choices, administrative agendas, institutional policies, and legislation) (3). Unlike a *moral dilemma* in which one is uncertain what ethical action to take, MoD is experienced by those who feel constrained from acting on their ethical judgment. Constraints are still recognized frequently as external, institutional ones (4). Internal constraints may be related to perceived powerless, lack of knowledge, increased moral sensitivity, or even lack of full understanding of a particular situation. It could also represent a lack of “moral courage” (5).

In 2006, Nathaniel extended the definition, highlighting the consequences of not acting according with ones’ moral judgment and be participating in perceived moral wrongdoing (6). The word *perceived* is very crucial, since we may feel strongly that an action is unethical while a colleague may feel just as strongly the opposite. It is well known that MoD in pediatric intensive care (PICU) can be linked to aggressive treatment, witnessing repeated suffering, futile care, and high levels of chronic disability post discharge and may be aggravated by work environment issues such as power imbalances, improper communication, decision-making conflicts, unrealistic expectations, lack of resources or personnel, and a high index of medical errors (2, 7) Corley and colleagues have developed a scale (MDS), containing 20 clinical situations to assess the frequency to which MoD occurs, as well as the intensity of the feeling (8) This scale, now on its second version, has been utilized in several studies (4, 5), including some in the PICU environment (9). As MoD has been more thoroughly investigated, discussion about the topic has become more prominent in the bioethics literature, with several journal issues being fully dedicated to the theme (10, 11).

## Why Does Moral Distress Occur in Intensive Care Units?

In this high-tension, rapidly changing environment, team work is paramount – and it is the way we operate (12). Indeed, the best clinical outcomes in critical care are correlated with a “team” approach (13, 14). Intrateam discordance, however, has been identified as a significant factor related to MoD among all health-care disciplines (15). Team dynamics, such as power imbalances (e.g., the lack of inclusion of the bedside nurse in overall decision-making), silencing (e.g., pressure to refrain from raising ethical concerns or voicing doubt about desired outcomes), “professional tribalism” (16), lack of trust in existing systems for ethical dialogue and patient management (e.g., worry that the “ethics people” are there to protect the organization, not patients or staff), not only affect individual PICU team member’s behaviors but also can heighten MoD. Poor team communications and lack of provider continuity have also been found as reasons for MoD among the PICU team (17, 18).

In our narrative inquiry study of the MoD of PICU teams, we have found that PICU staff participants identified lack of organizational support as an important source for MoD in situations of interdisciplinary conflict (19, 20). While research indicates that genuine dialog among the team members regarding ethically difficult situations is much desired (18, 21), the sheer number of personnel necessary to provide 24-h care for a PICU patient makes such dialog a logistical challenge (13). How can it be ensured that all staff involved in a particular patient’s care are included in key discussions or even kept informed, in a timely way, of the ethical concerns being raised?

Finding answers to such logistical challenges is a worthwhile endeavor. The consequences of MoD are significant for any intensive care unit, at the personal, the team, and the institutional level. Conflicting views related to life-sustaining treatment deeply impact staff members. Physicians, for instance, can become detached, and their future medical decisions compromised by such experiences (15). An experience of MoD can haunt some individuals for years, in what has been called moral residue (22). In fact, it has been found that MoD can be a reason for health-care staff to quit their position or even their practice entirely (4).

There is some evidence that the intensity of MoD can vary according to two factors: personal moral sensitivity and the moral climate in the organization (23). MoD may be an expression of sensitivity to the moral aspects of practice, an appreciation of vulnerability of patients, a simple reaffirmation of one’s values expressed in codes of ethics, or perhaps an acceptance of accountability and moral responsibility (15). Such recognition allows greater openness about the experience and does much to prevent a staff member being seen as simply unable to cope in the technologically driven, fast-paced intensive care environment.

## Why is Moral Distress in Vogue Now?

Lantos suggests that, in many areas of medicine today, there is a lack of consensus as what should be the best treatment for particular patients (24). As Morparia and colleagues noted, this lack of consensus continues to feed the controversy. Physician, in almost all cases, are equally divided in their choices (25). Parents have become – properly so – increasingly important in the determination of treatment choices for their children; this results at times in clashing beliefs and values between parents and health-care systems (26). In our study, participants’ stories suggest that professionals can be seriously distressed by taking part in treatments and/or care that they believe to be wrong for a particular patient or family (19, 20), especially when dealing with end-of-life decisions – a finding reported by others as well (27). In a recent study within an adult ICU, Dodek and colleagues found that MoD ultimately stems from three problems: uncertainty about who is in charge, cost-cutting schemes that affect patient care, and controversies about end-of-life (4, 28). An excellent review of MoD in neonatal and pediatric ICUs, by Sauerland and colleagues, concluded that situations causing the most intense distress were related to inadequate nurse staffing and perceived incompetent coworkers. The most frequently occurring distressing items were futile care and unsafe staffing (9).

Moral distress, however, becomes magnified when one feels that it is unsafe to voice one’s concern. A culture of silence can prevail to the extent that professionals know that raising any ethical concern will too easily label them as a troublemaker or as someone who is unable to “take the pressure.” The lack of authentic debriefing and/or ethical dialog that occurs within such a culture appears to be a major contributor to moral distress (15).

## How Can we Survive in the ICU?

Moral distress, like burnout, is a reality of our times. We have to find ways to meaningfully address it. Not to do so can mean that we will lose highly ethical physicians who find no recourse but to leave the job they love, forfeiting years of training and personal sacrifice (28). To prevent MoD, a culture of frank dialogue and good team communication is fundamental. In the PICU, finding the time and space for these to occur is an ongoing challenge.

## How Can we Address Moral Distress in the PICU?

Based on the current research and personal experience, the following are some practical ways that can help physicians and their teams to prevent or to address MoD:

1. *Recognize* that MoD can be an alarm signal raised by a conscientious person encountering an ethical problem and worried that something ethically wrong is going to happen (29).
2. *Give voice to the silence*: whenever possible, foster open and authentic discussion of ethical concerns from each person’s perspective, including health-care professionals, trainees, parents, families, and patients. All practitioners on the team, regardless of discipline or “professional ranking,” should be able to safely raise their concerns of conscience (30). A culture of ethical questioning should be endorsed by institutions, similar to the openness described in the airline industry for safety concerns (31).
3. *Reach for a “rapprochement*”: we need to find ways to support moving to reciprocal understandings among those involved in patient care, as proposed by Carnevale (32). Respect, trust, and honesty will be required for common ground to be found in difficult situations.
4. *Enhance effective communication among team members*. In the fast-paced PICU environment, “real time” team sharing of information regarding treatment options and decisions made between physicians and families is crucial (30, 33).
5. *Seek further ethics education*: ongoing education in health ethics is one way to evolve a shared language by which the team may address ethical concerns; it may allow a deeper understanding of what is at stake (34).
6. *Promote “venting/debriefing sessions”*: after difficult cases, such sessions allow for open conversations and analysis. These sessions, to be successful, need to be characterized by authenticity, compassion, regrouping, and validation (30). Such encounters are crucial for the well-being of the unit and the practitioners involved and should be supported (i.e., finances, resources, and training) by the institutions (15, 35).

At the personal, individual level, a general mindset that encompasses the following may be helpful:

1) *Nourish “moral courage”*: an ethical health-care professional will always need moral courage, i.e., be prepared to face tough decisions and confront the uncertainties associated with the resolve to *do the right thing* despite the consequences faced. This need for moral courage is especially true when the perceived constraints are within ourselves or when we face opposition within our own ranks. The courageous person accepts and assumes moral responsibility for the perceived consequences of his or her action; a physician may master fear without necessarily eliminating it (36).
2) *Seek peer support*: this type of sharing, with someone that can understand one’s struggles, has a good listening ear and acknowledges and validates what one is experiencing seems essential for survival in the profession (15).
3) *Take time off*: having resting periods, time offs, vacation, and long weekend getaways: these are necessary for one’s well-being. Physical activity, exercise, enjoying nature, and managing stress through mindfulness are strategies shown to increase the capacity to take new challenges with stride (1).
4) *Find another passion*: having another interest outside work, even one connected with the field, such as volunteering on medical missions, has been very rewarding for me. Having a favorite sport, a pet, or pursuit (e.g., mountain biking, skiing, gardening, and traveling) have been cited by many PICU colleagues as their way to recharge and continue on the job. Cultivating a spiritual life, within or outside an organized religion, has been found to be important to sustain one’s motivation and engagement. To focus on what really matters in the work I do – the children and their families – certainly helps me keep going.

There is much that can be done to make the PICU a more “morally habitable” place. Given the current levels of reported MoD, health administrators need to attend to this important workplace factor and ensure the support necessary for staff to address it. As practitioners, we ought to develop insight into our feelings and reactions, contribute to an open environment where team work is a healthy practice, and adopt a lifestyle that recharges us for the next difficult case.

## Author Contributions

The author confirms being the sole contributor of this work and approved it for publication.

## Conflict of Interest Statement

The author declares that the research was conducted in the absence of any commercial or financial relationships that could be construed as a potential conflict of interest.
